# A community-based isoniazid preventive therapy for the prevention of childhood tuberculosis in Ethiopia

**DOI:** 10.5588/ijtld.16.0471

**Published:** 2017-09-01

**Authors:** D. G. Datiko, M. A. Yassin, S. J. Theobald, L. E. Cuevas

**Affiliations:** * REACH Ethiopia, Hawassa, Southern Region, Ethiopia; † Liverpool School of Tropical Medicine, Liverpool, UK; ‡ The Global Fund to fight AIDS, Tuberculosis and Malaria, Geneva, Switzerland

**Keywords:** tuberculosis, children, preventive therapy, health extension workers, Ethiopia

## Abstract

**BACKGROUND:**

Although children in contact with adults with tuberculosis (TB) should receive isoniazid (INH) preventive therapy (IPT), this is rarely implemented.

**OBJECTIVE:**

To assess whether a community-based approach to provide IPT at the household level improves uptake and adherence in Ethiopia.

**METHODS:**

Contacts of adults with smear-positive pulmonary TB (PTB+) were visited at home and examined by health extension workers (HEWs). Asymptomatic children aged <5 years were offered IPT and followed monthly.

**RESULTS:**

Of 6161 PTB+ cases identified by HEWs in the community, 5345 (87%) were visited, identifying 24 267 contacts, 7226 (29.8%) of whom were children aged <15 years and 3102 (12.7%) were aged <5 years; 2949 contacts had symptoms of TB and 1336 submitted sputum for examination. Ninety-two (6.9%) were PTB+ and 169 had TB all forms. Of 3027 asymptomatic children, only 1761 were offered (and accepted) IPT due to INH shortage. Of these, 1615 (91.7%) completed the 6-month course. The most frequent reason for discontinuing IPT was INH shortage.

**CONCLUSION:**

Contact tracing contributed to the detection of additional TB cases and provision of IPT in young children. IPT delivery in the community alongside community-based TB interventions resulted in better acceptance and improved treatment outcome.

## Abstract (FR)

**CONTEXTE:**

Les enfants en contact avec des adultes atteints de tuberculose (TB) devraient recevoir un traitement préventif par isoniazide (IPT), mais ceci est rarement mis en œuvre.

**OBJECTIF:**

Evaluer si une approche basée en communauté fournissant l'IPT au niveau du foyer améliore la prise du traitement et l'adhérence en Ethiopie.

**MÉTHODES:**

Les contacts des adultes atteints de TB pulmonaire à frottis positif (TBP+) ont bénéficié d'une visiteà domicile et d'un examen par des agents de vulgarisation sanitaire (HEW). Les enfants asymptomatiques âgés de <5 ans ont reçu l'IPT et ont été suivis une fois par mois.

**RÉSULTATS:**

Des 6161 cas de TBP+ identifiés par les HEW au sein de la communauté, 5345 (87%) ont été visités, ce qui a permis d'identifier 24 267 contacts : 7226 (29,8%) contacts ont été des enfants âgés de <15 ans et 3102 (12,7%) d'entre eux avaient <5 ans ; 2949 contacts avaient des symptômes de TB et 1336 ont soumis unéchantillon de crachats pour examen. En tout, 92 (6,9%) avaient une TBP+ et 169 une TB de forme variée. Sur 3027 enfants asymptomatiques, seulement 1761 ont été invités à prendre l'IPT (et l'ont accepté) en raison de ruptures de stock d'isoniazide. Parmi ces derniers, 1615 (91,7%) ont achevé les 6 mois de traitement. La raison la plus fréquente de suspendre l'IPT a été la rupture de stock d'isoniazide.

**CONCLUSION:**

La recherche des contacts a contribué à la détection de cas supplémentaires de TB et à la fourniture de l'IPT aux jeunes enfants. La fourniture de l'IPT dans la communauté parallèlement à des interventions communautaires relatives à la TB a abouti à une meilleure acceptation du traitement et à un meilleur résultat.

## Abstract (SP)

**MARCO DE REFERENCIA:**

Los niños que están en contacto con adultos con diagnóstico de tuberculosis (TB) deberían recibir el tratamiento preventivo con isoniazida (IPT), pero esta norma no suele aplicarse.

**OBJETIVO:**

Analizar si una estrategia de dispensación comunitaria del IPT en los hogares mejoraría la aceptación y la adherencia a este tratamiento en Etiopía.

**MÉTODOS:**

Los trabajadores de extensión sanitaria (HEW) visitaron en su hogar a los contactos de los adultos con diagnóstico de TB pulmonar a baciloscopia positiva (TBP+). Se propuso a los niños de edad <5 años el IPT y se practicó un seguimiento mensual.

**RESULTADOS:**

De los 6161 casos de TBP+, los HEW visitaron a 5345 casos (87%) en la comunidad y reconocieron 24 267 contactos. De estos contactos, 7226 (29,8%) eran niños <15 años de edad y 3102 (12,7%) tenían <5 años de edad. De los contactos, 2949 refirieron síntomas indicativos de TB y 1336 aportaron muestras de esputo para examen. Se detectaron 92 casos de TBP+ (6,9%) y 169 casos con todas las formas de TB. De los 3027 niños asintomáticos, se ofreció el IPT solo a 1761 (y lo aceptaron) debido a la escasez de isoniazida. De estos niños, 1615 completaron la pauta de tratamiento de 6 meses (91,7%). La causa más frecuente de abandono del IPT fue también la escasez del medicamento.

**CONCLUSIÓN:**

La localización de contactos contribuyó a la detección de casos adicionales de TB y mejoró la provisión del IPT a los niños pequeños. La dispensación comunitaria del IPT aunada a otras intervenciones contra la TB en la comunidad tuvo como resultado una mayor aceptación del tratamiento y mejores desenlaces terapéuticos.

CHILDREN IN CONTACT with adults with smear-positive pulmonary tuberculosis (PTB+)have a high risk of infection and disease progression; contact investigation is therefore critical for diagnosing additional cases and preventing vulnerable individuals from progressing from infection to overt disease. Nearly all National TB Control Programmes (NTPs) recognise this risk and recommend screening contacts, especially children, for the presence of symptoms. According to international recommendations, symptomatic contacts should be investigated for active TB, whereas asymptomatic children aged <5 years should receive isoniazid (INH) preventive therapy (IPT).^1^

Although IPT reduces the risk of disease progression,^1^ very few NTPs in low- and middle-income countries (LMICs) implement this recommendation.^2^,^3^ This is because INH is often unavailable, staff have the perception that the risk of disease progression is low, that monotherapy promotes the risk of drug resistance if active TB is not excluded and due to reluctance to take on additional work.^4^,^5^ Parents are also reluctant to give pills to their asymptomatic children and adhere poorly to the 6-month course.^3^,^6^,^7^

In 2010, we implemented a novel community-based approach to enhance TB case finding and treatment outcome in southern Ethiopia. This project trains female health extension workers (HEWs) to identify individuals with symptoms of TB, collect sputum specimens and prepare and fix smears for examination in ‘kebeles’ (equivalent to a village, with an average population of 5000 people).^8^ HEWs are supported by supervisors who transport the smears to the nearest diagnostic laboratory and bring the drugs to initiate treatment at home or at the kebele health post. Contacts of adult TB cases are required to be screened for TB. However, as it was unlikely contacts would attend the health facilities or bring their children for screening,^8^ we initiated contact tracing and the provision of IPT in the community through HEWs. We report the acceptability of and adherence to IPT among children in contact with adults with PTB+ identified and managed in the community by HEWs.

## METHODS

This was a prospective community-based cohort study of individuals residing in a household of adults who had been diagnosed with PTB+ in the Sidama Zone, in the Southern Nations Nationalities and Peoples' Regional State (SNNPR) of Ethiopia. The project covered the entire zone, which has a population of 3.2 million residing in 19 rural districts and two town administrations. In this zone, three hospitals and 104 health centres provide health services to 524 rural and 39 urban kebeles.^9^ The project was implemented under the Ethiopian Health Extension Programme (HEP), which has been providing health services at the village level since 2003. The HEP trains women who have completed secondary education for 1 year; HEWs are salaried cadres responsible for routinely conducting household visits to implement 16 basic health packages.^10^

Adults with PTB+ reported here were diagnosed between May 2011 and March 2013, as previously described. Briefly, individuals with cough of ⩾ 2 weeks were identified by HEWs during routine household visits. Symptomatic individuals were requested to provide two sputum specimens over 2 consecutive days. Smears were prepared and fixed by the HEWs and transported by supervisors to the nearest laboratory. If the smear was positive, the supervisor brought the anti-tuberculosis drugs to the village and initiated treatment at home or at the health post, depending on the patient's preference.^8^

All household members were considered contacts of the index case. The index cases were asked to list all household members and their ages at the time the supervisor or HEW visited the household to disclose the diagnosis of PTB+ and initiate treatment. This list was used to ask about the presence of symptoms suggestive of TB among contacts. Houses of index cases reporting symptomatic household contacts not present at the time of the first visit were revisited to examine the contacts. However, asymptomatic contacts who were absent from the household at the time the list was prepared were not systematically revisited.

Presumptive cases among contacts were defined as individuals who reported having cough of ⩾2 weeks with or without chest pain, shortness of breath, fever, weight loss, failure to thrive or night sweats. Presumptive cases able to expectorate were requested to submit sputum specimens. Children unable to produce sputum were referred to the nearest health facilities for further clinical examination and chest X-ray (CXR). Transport subsidies were provided as needed for household members who could not afford to travel to ensure they were able to access the services. TB cases diagnosed among symptomatic contacts were provided treatment in the same manner as the index cases. Children considered to have other infections were given broad-spectrum antibiotics and were followed up by health facilities.

Asymptomatic children aged <5 years were offered 6 months of INH at a daily dose of 5 mg/kg. Children were not tested with the tuberculin skin test or interferon-gamma release assays, as these are not locally available and are not indicated in the Ethiopian TB guidelines.^10^ Parents and guardians of the children were instructed to break the tablets into two (or four) pieces to provide the closest approximate dose possible. Parents of children aged <2 years or unable to swallow tablets were advised to crush the pieces into a powder. Parents were advised on the symptoms of TB, the importance, purpose and side effects of IPT and to seek the HEWs for advice if the child developed symptoms or side effects. Adult contacts received health education about TB, its transmission, symptoms and what to do if symptoms developed in the future.

Index cases receiving anti-tuberculosis drugs and children receiving IPT were followed by HEWs and volunteer TB treatment supporters. Follow up comprised monthly home visits or visits at the health post at the same time as the index case was receiving anti-tuberculosis treatment. During each visit, parents/guardians were asked by the HEWs about the presence of symptoms, adverse effects and adherence. Parents of children with minor side effects were advised to continue IPT and to immediately report any changes. Children with major IPT side effects or TB symptoms were told to discontinue IPT and were referred to the nearest hospital, where they were investigated and monitored. ‘Refusal to accept IPT’ was defined as a parent refusing to initiate IPT. ‘IPT discontinuation’ was defined as a child initiating IPT but stopping the medication for >2 months continuously in the absence of side effects or on medical advice.

Parents or guardians were informed about the importance of IPT and adherence, and children took their medication under their supervision. Parents or guardians visited their local health posts for refills and discussed any concerns about the medication, side effects and TB-related symptoms with HEWs or supervisors. Monthly meetings between HEWs, supervisors and health centre staff were held to discuss progress in the implementation of IPT in the community. Children receiving IPT were registered in health post IPT registers, and data were updated monthly and at the time of drug refills.

Semi-structured questionnaires were used for symptom screening among household contacts. TB and IPT registers were used to collect data related to diagnosis for active TB and IPT outcomes. Questionnaires were checked for completeness and consistency; data were entered by a data officer into Excel^™^ (MicroSoft, Redmond, WA, USA) and exported to SPSS for Windows^™^ 20 (Statistical Package for the Social Sciences, Armonk, NY, USA). The main outcomes of the study were the number of children who completed 6-month IPT and the number who discontinued or were lost to follow-up. Secondary outcomes were the number of children initiating IPT who developed minor and major side effects and the number of children diagnosed with TB or who died during the 6-month follow-up. We conducted a univariate analysis for categorical variables. *P* < 0.05 was considered statistically significant. Children aged <5 years were categorised by age group to identify risk factors for IPT discontinuation.

The Federal Ministry of Health of Ethiopia, Addis Ababa, and the Southern Regional Health Bureau, Hawassa, Ethiopia, provided written support for the implementation of the study. The study protocol was submitted for consideration to the Liverpool School of Tropical Medicine Ethics Committee, Liverpool, UK. The Committee waived the need for ethics approval as it considered the project was implementing internationally accepted treatment. Parents were informed that the service was part of national TB guidelines and that data monitoring was required to document the proportion of children who completed the treatment.

## RESULTS

A total of 10 066 PTB+ cases were notified from the Sidama Zone from November 2010 to March 2013. Of these, 6161 (61%) had been identified by HEWs. Contact tracing was mostly conducted for index cases identified by HEWs. A total of 5345 index cases (53%) had their contacts enumerated, generating a list of 24 267 household contacts. Of these, 7226 (29.8%) were children aged <15 years, including 3102 (12.7%) children aged <5 years. A total of 2949 (12.2%) of the 24 267 contacts had symptoms of TB, including 523/7226 (7.2%) children aged <15 years and 75/3102 (2.4%) children aged <5 years. Of the 2949 symptomatic contacts, 1336 (45%) submitted sputum for examination.

Ninety-two (6.9% of 1336 symptomatic contacts with smear examination) had smear-positive PTB and 169 (12.7%) had TB all forms (Table 1). The median age of the 75 symptomatic children aged <5 years was 39 months (range 1–72) compared with 47 months for the 1730 asymptomatic children aged <5 years (31 had data for age missing).

**Table 1:**
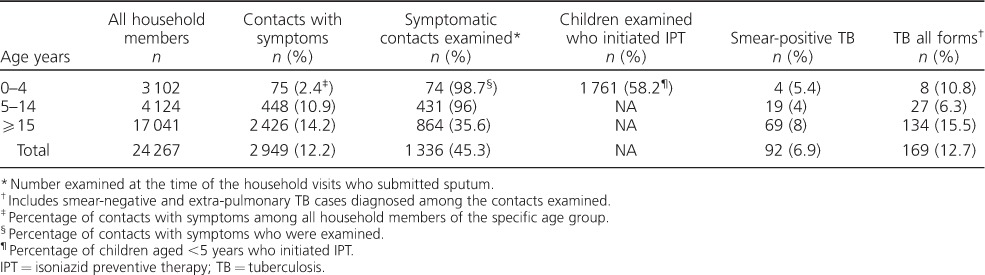
The yield of household contact screening and IPT provision in southern Ethiopia

Only 472 (93.4%) of the 523 symptomatic children aged <15 years who were examined had symptoms recorded, compared with 100% of the 864 symptomatic adults who were examined. The most common symptoms identified in these children were cough (66.3%), fever (63.8%), shortness of breath (55.7%) and constitutional symptoms, including night sweats, loss of appetite and weight loss. Only a few children had haemoptysis. These frequencies were similar to those recorded among symptomatic adults, except for cough, which was more common among adults (*P* < 0.01, Table 2).

**Table 2:**
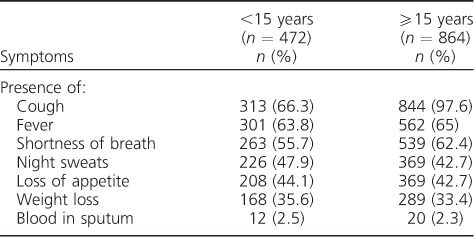
Most frequent symptoms reported among contacts of index pulmonary tuberculosis cases who submitted sputum (n = 1336)

Of the 3027 asymptomatic children aged <5 years who were eligible for IPT, 1761 (58%) were offered IPT (males 888, 50.4%; females 867, 49.4%; sex data were missing in 6). The remaining 1266 (42% of the 3027 eligible) were not offered IPT due to INH shortage. All parents who were offered IPT accepted the therapy.

In total, 1574 (89.3%) completed the 6-month course of IPT (Table 3). Infants appeared to be more likely to discontinue IPT than those aged 1–2 years and 3–5 years (89.0% vs. 90.8% and 92.1%, respectively), and to do so within 1 month of prophylaxis initiation. However, the frequencies were small and did not reach statistical significance. The proportion of children adhering to IPT at monthly intervals by age is shown in the Figure (age was missing for 103 children). The most frequent reasons for discontinuing IPT were INH shortage (*n* = 133), the death of a parent or the index case (*n* = 4) and side effects (*n* = 3). The main reported side effects of IPT included nausea and vomiting, which resolved spontaneously. One child developed symptoms compatible with hepatitis and required hospitalisation. As the hospitals do not have the diagnostic capacity to exclude other common causes of jaundice, such as viral hepatitis, IPT was discontinued in this child as a precaution. A further six patients developed symptoms compatible with TB, and underwent CXR. Three cases (0.17%) were diagnosed as having TB and 3 (0.17%) died due to other medical illnesses (2 were malnourished and were considered to have sepsis and 1, whose parents were infected with the human immunodeficiency virus, developed abdominal distension and severe oedema).

**Table 3:**
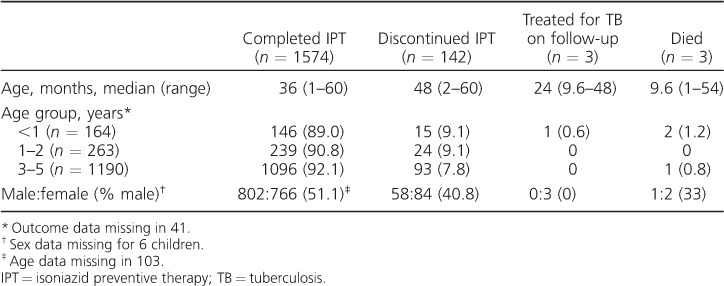
Characteristics of asymptomatic children aged <5 years who received IPT (n = 1761) ^*^

**Figure:**
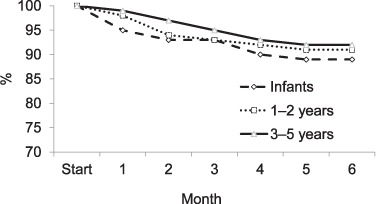
Proportion of children who adhered to IPT by age group and month of treatment. IPT = isoniazid preventive therapy.

## DISCUSSION

Despite the universal recognition of the value of the systematic screening and provision of IPT among children in contact with adults with TB,^11–14^ very few NTPs in LMICs implement this service at the health facility level,^12^,^15^,^16^ and even fewer NTPs are able to provide the service at the community level in poor, remote and rural communities. Reasons for not implementing contact investigation and IPT range from lack of prioritisation by NTPs, logistical problems in securing INH supplies, the time required to initiate and monitor IPT, staff perception that the risk of disease progression was minimal and the risk of developing drug resistance.^12^,^17–20^

We report our experience in implementing IPT at the community level among children within the framework of the Ethiopian Health Extension Programme. Children and adults exposed to adults with pulmonary TB were visited at home and questioned about the presence of symptoms; symptomatic individuals were asked to provide sputum samples. Of the 7226 children aged <15 years, 523 were reported to be symptomatic and 35 had TB. All parents with children aged <5 years visited by HEWs who were offered IPT accepted the therapy and attained high adherence, with >90% of the children completing the 6-month course. Despite the acceptability of the approach, an important shortcoming of the intervention was the major logistical problems faced in procuring INH despite a close partnership with the NTP. Before the intervention, the NTP had only implemented IPT at the health facility level and on a small scale, and the relatively large scale of our project led to major national procurement problems. The government also requested proxy registrations of the children at the nearest health centre before releasing the INH. This resulted in bottlenecks and a protracted process to release the drugs to the community, which took several months to resolve.

Our study had methodological limitations that must be explained to facilitate interpretation. The intervention was conducted under operational conditions, with the purpose of developing a system to provide TB services close to the community. As such, we were limited by the amount of information that could be collected to avoid overburdening the system. We were unable to obtain sputum from about 55% of the contacts who initially reported having symptoms. This was due to contacts being unable to expectorate, having no cough or because the symptoms resolved before the HEW had examined them. Furthermore, although children who could not expectorate were referred for CXR, not all parents were able to attend the clinic and some TB cases were likely missed. A further limitation is the underrepresentation of selected age groups, as only 29.8% of the contacts identified were children. This is in contrast to the national population statistics, which estimate that 44% of the population is aged <15 years. This deficit may reflect the working pattern of the HEWs, as household visits were conducted during working hours, and children may have been missed due to behaviour patterns, such as herding and school activities. Finally, child-friendly formulations, such as those that have been recently developed, would have simplified the delivery of prophylaxis, but these were not available at the time of the study.^21^ The INH dose used (5 mg/kg) might also have been too low, as the World Health Organization increased the recommended dose to 7–10 mg/kg after the study had been implemented for 2 years.

Despite these limitations, the approach presented here suggests that partnership with HEWs to provide community-based screening of contacts and IPT can achieve a high degree of acceptance and adherence. To our knowledge, this is the highest adherence level ever reported from this continent,^7^,^22^ suggesting that a large component of the lack of acceptability and adherence reported by most studies may be the poor accessibility of the service.

Although these results are encouraging, a major precondition for the provision of the service without incurring major health service expenses was the availability of community-based services, with the deployment of multipurpose HEWs at the village level. As these cadres are selected from and by the community, they are likely to remain in the village and have trusting relationships with the community. Several evaluations of the programme have demonstrated their impact in other health areas,^23^,^24^ and the acceptability of TB packages.^25^ A further constraint is the inherent ineffectiveness of contact investigation as a method of early detection of TB cases, as secondary TB cases typically develop after a lag time of several weeks in the first 2 years after infection. Asymptomatic contacts could thus develop TB at a later time, and fewer secondary cases will be detected if contacts are only interviewed for symptoms immediately after the diagnosis of the index case. Furthermore, symptom-based screening can miss many TB cases, as it is increasingly recognised that this method has poor sensitivity. Children receiving IPT may therefore be re-exposed to TB when older members of the household develop TB, and parents need to be aware of the need to maintain vigilance for symptoms for a long time.

We had reported earlier that very few children in contact with adult TB cases initiating IPT completed the 6-month prophylaxis in the SNNPR,^7^ and that adherence was poor, with very few parents continuing beyond the first few weeks. Contact investigations at health facilities are often conducted days or weeks after initiation of treatment of the index case and once the treatment has been established. This delay separates the processes of treating the patient and preventing further disease in the family, potentially contributing to lower IPT uptake and adherence. In the present study, we observed that initiating anti-tuberculosis treatment and IPT together creates a better mind imprint of the importance of adherence, and that it is convenient for the parents to collect the drugs at the same time as receiving treatment. This temporal association could be one of the reasons for the high adherence rates attained, in addition to the client-friendly characteristics of the community-based approach implemented, but we were unable to separate these factors.

In conclusion, this innovative intervention demonstrates that implementing community-based contact tracing and IPT provision among children is feasible under programme settings and can attain high adherence and completion rates in the Ethiopian context.

## References

[1] Marais B J, Ayles H, Graham S M, Godfrey-Faussett P. Screening and preventive therapy for tuberculosis. Clin Chest Med 2009; 30: 827– 846. 19925970 10.1016/j.ccm.2009.08.012

[2] World Health Organization. Guidance for national tuberculosis programmes on the management of tuberculosis in children. Geneva, Switzerland: WHO, 2006: p 41. 24999516

[3] Gomes V F, Wejse C, Oliveira I, Adherence to isoniazid preventive therapy in children exposed to tuberculosis: a prospective study from Guinea-Bissau. Int J Tuberc Lung Dis 2011; 15: 1637– 1643. 22118171 10.5588/ijtld.10.0558

[4] van Zyl S, Marais B J, Hesseling A C, Gie R P, Beyers N, Schaaf H S. Adherence to anti-tuberculosis chemoprophylaxis and treatment in children. Int J Tuberc Lung Dis 2006; 10: 13– 18. 16466031

[5] Marais B J, Gie R P, Schaaf H S, Beyers N, Donald P R, Starke J R. Childhood pulmonary tuberculosis: old wisdom and new challenges. Am J Respir Crit Care Med 2006; 173: 1078– 1090. 16484674 10.1164/rccm.200511-1809SO

[6] Bibi H, Weiler-Ravell D, Shoseyov D, Feigin I, Arbelli Y, Chemtob D. Compliance to treatment of latent tuberculosis infection in a region of Israel. Isr Med Assoc J 2002; 4: 13– 16. 11802301

[7] Garie K T, Yassin M, Cuevas L E. Lack of adherence to isoniazid chemoprophylaxis in children in contact with adults with tuberculosis in Southern Ethiopia. PLOS ONE 2011; 6: e26452. 22069451 10.1371/journal.pone.0026452 PMC3206033

[8] Yassin M A, Datiko D G, Tulloch O, Innovative community-based approaches doubled tuberculosis case notification and improve treatment outcome in Southern Ethiopia. PLOS ONE 2013; 8: e63174. 23723975 10.1371/journal.pone.0063174 PMC3664633

[9] Dangisso M H, Datiko D G, Lindtjorn B. Trends of tuberculosis case notification and treatment outcomes in the Sidama Zone, southern Ethiopia: ten-year retrospective trend analysis in urban-rural settings. PLOS ONE 2014; 9: e114225. 25460363 10.1371/journal.pone.0114225 PMC4252125

[10] Ethiopia Federal Ministry of Health. Tuberculosis, leprosy and TB/HIV prevention and control programme. 4th ed. Addis Ababa, Ethiopia: Ethio Tiku Printing Press, 2006.

[11] Aminzadeh Z, Asl R T. A six months follow-up on children less than 6 years old in contact with smear positive tuberculosis patients, Varamin City, Tehran, Iran. Int J Prev Med 2011; 2: 79– 81. 21603012 PMC3093776

[12] Rutherford M E, Hill P C, Triasih R, Sinfield R, van Crevel R, Graham S M. Preventive therapy in children exposed to Mycobacterium tuberculosis: problems and solutions. Trop Med Int Health 2012; 17: 1264– 1273. 22862994 10.1111/j.1365-3156.2012.03053.x

[13] Getahun H, Sculier D, Sismanidis C, Grzemska M, Raviglione M. Prevention, diagnosis, and treatment of tuberculosis in children and mothers: evidence for action for maternal, neonatal, and child health services. J Infect Dis 2012; 205 Suppl 2: S216– S227. 22448018 10.1093/infdis/jis009

[14] Hill P C, Rutherford M E, Audas R, van Crevel R, Graham S M. Closing the policy-practice gap in the management of child contacts of tuberculosis cases in developing countries. PLOS Med 2011; 8: e1001105. 22022234 10.1371/journal.pmed.1001105 PMC3191150

[15] Shivaramakrishna H R, Frederick A, Shazia A, Isoniazid preventive treatment in children in two districts of South India: does practice follow policy? Int J Tuberc Lung Dis 2014; 18: 919– 924. 25199005 10.5588/ijtld.14.0072 PMC4589200

[16] Pothukuchi M, Nagaraja S B, Kelamane S, Tuberculosis contact screening and isoniazid preventive therapy in a South Indian district: operational issues for programmatic consideration. PLOS ONE 2011; 6: e22500. 21799875 10.1371/journal.pone.0022500 PMC3142154

[17] van Soelen N, du Preez K, van Wyk S S, Does an isoniazid prophylaxis register improve tuberculosis contact management in South African children? PLOS ONE 2013; 8: e80803. 24339884 10.1371/journal.pone.0080803 PMC3858233

[18] Van Wyk S S, Hamade H, Hesseling A C, Beyers N, Enarson D A, Mandalakas A M. Recording isoniazid preventive therapy delivery to children: operational challenges. Int J Tuberc Lung Dis 2010; 14: 650– 653. 20392361

[19] van Wyk S S, Reid A J, Mandalakas A M, Operational challenges in managing isoniazid preventive therapy in child contacts: a high-burden setting perspective. BMC Public Health 2011; 11: 544. 21740580 10.1186/1471-2458-11-544 PMC3150266

[20] Rutherford M E, Ruslami R, Maharani W, Adherence to isoniazid preventive therapy in Indonesian children: a quantitative and qualitative investigation. BMC Res Notes 2012; 5: 7. 22221424 10.1186/1756-0500-5-7 PMC3287144

[21] World Health Organization. World's first child-friendly TB medicines in correct doses. Geneva, Switzerland: WHO, 2016 http://www.who.int/tb/features_archive/FDC_formulation_ launch/en/. Accessed May 2017.

[22] Marais B J, van Zyl S, Schaaf H S, van Aardt M, Gie R P, Beyers N. Adherence to isoniazid preventive chemotherapy: a prospective community based study. Arch Dis Child 2006; 91: 762– 765. 16737993 10.1136/adc.2006.097220 PMC2082929

[23] Teklehaimanot H D, Teklehaimanot A. Human resource development for a community-based health extension program: a case study from Ethiopia. Human Res Health 2013; 11: 39. 10.1186/1478-4491-11-39 PMC3751859 23961920

[24] Wakabi W. Extension workers drive Ethiopia's primary health care. Lancet 2008; 372: 880. 18795419 10.1016/s0140-6736(08)61381-1

[25] Datiko D G, Yassin M A, Tulloch O, Exploring providers' perspectives of a community based TB approach in Southern Ethiopia: implication for community based approaches. BMC Health Serv Res 2015; 15: 501. 26553340 10.1186/s12913-015-1149-9 PMC4638085

